# Subcutaneous vitamin B12 administration using a portable infusion pump in cobalamin-related remethylation disorders: a gentle and easy to use alternative to intramuscular injections

**DOI:** 10.1186/s13023-021-01847-9

**Published:** 2021-05-12

**Authors:** Amelie S. Lotz-Havla, Katharina J. Weiß, Katharina A. Schiergens, Theresa Brunet, Jürgen Kohlhase, Stephanie Regenauer-Vandewiele, Esther M. Maier

**Affiliations:** 1 Department of Inborn Errors of Metabolism, Dr. von Hauner Children’s Hospital, Ludwig-Maximilians-University, Lindwurmstr. 4, 80337 Munich, Germany; 2 Institute of Human Genetics, Technische Universität München, Trogerstr. 32, 81675 Munich, Germany; 3 SYNLAB Center for Human Genetics, Heinrich-von-Stephan-Str. 5, 79100 Freiburg, Germany

**Keywords:** Remethylation disorder, Cobalamin, Hydroxycobalamin, Subcutaneous catheter system, Subcutaneous infusion, Infusion pump

## Abstract

**Background:**

Cobalamin (cbl)-related remethylation disorders are a heterogeneous group of inherited disorders comprising the remethylation of homocysteine to methionine and affecting multiple organ systems, most prominently the nervous system and the bone marrow. To date, the parenteral, generally intramuscular, lifelong administration of hydroxycobalamin (OHCbl) is the mainstay of therapy in these disorders. The dosage and frequency of OHCbl is titrated in each patient to the minimum effective dose in order to account for the painful injections. This may result in undertreatment, a possible risk factor for disease progression and disease-related complications.

**Results:**

We describe parenteral administration of OHCbl using a subcutaneous catheter together with a portable infusion pump in a home therapy setting in four pediatric patients with remethylation disorders, two patients with cblC, one patient with cblG, and one patient with cblE deficiency, in whom intramuscular injections were not or no longer feasible. The placement of the subcutaneous catheters and handling of the infusion pump were readily accomplished and well accepted by the patients and their families. No adverse events occurred. The use of a small, portable syringe driver pump allowed for a most flexible administration of OHCbl in everyday life. The concentrations of total homocysteine levels were determined at regular patient visits and remained within the therapeutic target range. This approach allowed for the continuation of OHCbl therapy or the adjustment of therapy required to improve metabolic control in our patients.

**Conclusions:**

Subcutaneous infusion using a subcutaneous catheter system and a portable pump for OHCbl administration in combined and isolated remethylation disorders is safe, acceptable, and effective. It decreases disease burden in preventing frequent single injections and providing patient independence. Thus, it may promote long-term adherence to therapy in patients and parents.

## Background

Cobalamin (cbl)-related remethylation disorders comprise a heterogeneous group of inherited disorders affecting the remethylation of homocysteine to methionine. In different ways, they all lead to a deficient activity of methionine synthase: decreased function of the enzyme protein itself (cblG; OMIM #250940), decreased function of the reactivating enzyme methionine synthase reductase (cblE; OMIM #236270), or impaired supply of the cofactor methylcobalamin (cblC; OMIM #277400, cblD; OMIM #277410, cblF; OMIM #277380, cblJ; OMIM #614857) [1, 2]. Markedly elevated concentrations of total homocysteine (tHcy) in blood are the biochemical hallmark of cobalamin-related remethylation disorders. Some disorders of intracellular cobalamin metabolism (cblC, cblD-MMA/Hcy, cblF, and cblJ) do not only compromise the synthesis of methylcobalamin, but also the synthesis of adenosylcobalamin, cofactor of the enzyme methylmalonyl-CoA mutase, leading to elevated concentrations of both tHcy and methylmalonic acid (MMA). These disorders are referred to as combined remethylation disorders [2, 3].

The clinical signs and symptoms of cobalamin-related remethylation disorders may vary considerably and affect multiple organ systems, most prominently the nervous system and the bone marrow. They include megaloblastic anemia, lethargy, failure to thrive, microcephaly, brain abnormalities, developmental delay, intellectual deficit, seizures, and retinopathy. Most patients present during the neonatal period or in early infancy. However, juvenile or adult onset manifestations characterized mainly by ataxia, dementia, and psychosis have been described [2, 4].

The treatment of cobalamin-related remethylation disorders aims to reduce tHcy and—in combined defects—to normalize MMA in order to improve clinical features [2]. To achieve this, the administration of hydroxycobalamin (OHCbl) is the mainstay of therapy [2] with the parenteral route being strongly recommended [5–8]. The long-term intravenous (IV) application of OHCbl is impracticable for the patients. Hence, cobalamin-related remethylation disorders are usually treated by intramuscular (IM) injections at an individually titrated, minimum effective dose. The required frequency of injections ranges between daily and weekly [2]. However, the frequent and painful IM injections present a great burden for patients and their families, and subcutaneous (SQ) OHCbl administration has been suggested as an alternative parenteral route [9]*.* Its efficacy, though, is still debated [2, 4, 10, 11].

Within the last years, SQ infusion of an increasing number of drugs has been described to have favorable outcomes with regards to effectiveness, safety, acceptability, and efficiency in the pediatric and adult population [12].

We implemented the SQ infusion of OHCbl using a SQ catheter system together with a small, portable pump in four patients with cobalamin-related remethylation disorders in whom frequent IM injections were not or no longer feasible. We wish to raise awareness for this easy to use access and route of administration.

## Results

### Patient 1

**Fig. 1:**
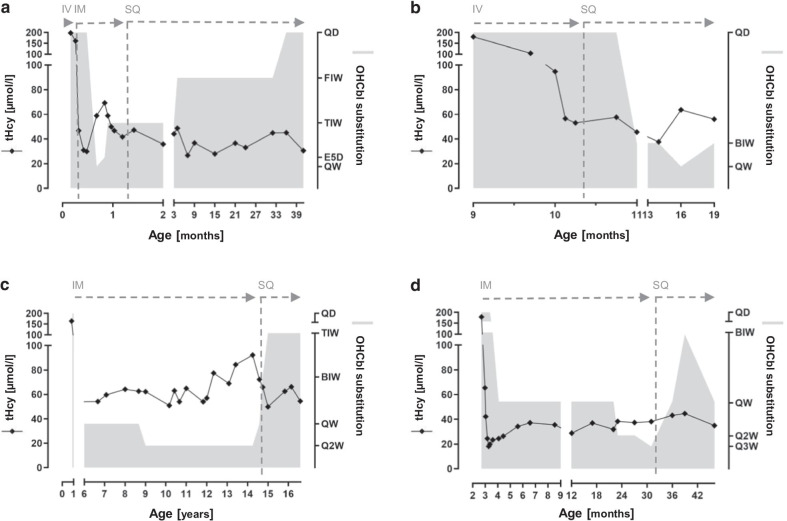
Metabolic control in dependence of OHCbl substitution. **a** Patient 1, **b** patient 2, **c** patient 3, and **d** patient 4. Concentrations of total homocystein (tHcy) in plasma [µmol/l] over the time are depicted by a rhombus. 1 mg OHCbl was injected IV (intravenous), IM (intramuscular) or SQ (subcutaneous), respectively, with varying frequency, demonstrated by the grey area (*QW* weekly, *Q2W* every 2 weeks, *Q3W* every 3 weeks, *BIW* twice a week, *TIW* three times a week, *FIW* five times a week, *QD* daily, *E5D* every 5 days)

Patient 1, a girl, was born at 39 + 2 weeks of gestation (birth weight 2310 g). She is the first child (G1P1) of healthy, non-consanguineous parents of Caucasian origin. Postnatal adaptation was uneventful. Dried blood spots for newborn screening were collected at 35 h of age and revealed slightly elevated concentrations of propionylcarnitine (8.9 µmol/l; cut-off < 5.9) and MMA (59.5 µmol/l; cut-off < 5), whereas the concentration of methionine (7 µmol/l; cut-off > 8) was decreased, and 3-hydroxy-propionic acid (26.7 µmol/l; cut-off < 30) was normal. The patient was transferred to our metabolic center. Clinical examination revealed feeding problems and muscular hypotonia. Confirmation testing at day 5 demonstrated markedly elevated concentrations of MMA (139,500 nmol/l; reference range 73–271), tHcy (198 µmol/l; reference range < 12), and propionylcarnitine (13.7 µmol/l; reference range < 1.4) in plasma. Analysis of organic acids in urine revealed increased excretions of MMA (2576 mmol/mol creatinine; reference range < 3.7), 3-hydroxy-propionat, and methylcitrate. The concentrations of ammonia (82 µmol/l; reference range < 110), lactate (1.7 mmol/l; reference range < 2.1), vitamin B12 (1780 pg/ml; reference range 197–771), and folic acid (19.8 ng/ml; reference range 3.9–26.8) were unremarkable. These findings are highly indicative for an inborn error of cobalamin metabolism, and supplementation of OHCbl 1 mg IV, folic acid 20 mg PO, betaine 250 mg/kg PO, and initially methionine 25 mg PO per day were started. With this treatment, tHcy and MMA in plasma declined to tHcy 47 µmol/l and MMA 12,360 nmol/l on day 10 of life (Fig. 1a). The diagnosis of cblC deficiency was confirmed by sequence analysis of the *MMACHC* gene. Two variants, the canonical splice site variant c.81 + 1G > T, p.? and a deletion of exon 4, were identified in compound heterozygous state. Both variants had not previously been described and were classified as pathogenic according to the American College of Medical Genetics (ACMG) guidelines [13]. The variant c.81 + 1G > T is predicted to disrupt the splice donor site of exon 1 as demonstrated for the known pathogenic variant c.81 + 1G > A [14]. The deletion of exon 4 is predicted to result in a functional loss of the enzyme.

Within the following weeks, treatment was adjusted based on tHcy concentrations in plasma (Fig. 1a). Treatment with methionine was stopped, betaine was continued with 250 mg/kg PO and folic acid with 10 mg PO per day. OHCbl was changed from IV to IM administration at day 8 of life. To achieve tHcy concentrations as low as possible (< 40 µmol/l), the administration of OHCbl (1 mg) was required every other day. The IM injections were painful for the newborn and went along with an enormous psychological stress for the mother. The mother was inclined to refuse further IM therapy. To reduce the burden of frequent injections, we started the SQ administration of OHCbl using a SQ catheter and a portable syringe driver pump as described in the methods section. The placement of the small SQ catheter was well tolerated and could be executed by the parents allowing for home therapy. The slow, pump-controlled delivery of OHCbl over 1 h prevented the pain induced by the swift delivery of the medication in IM or SQ injections. No complications such as local infections at the catheter site occurred. The use of a portable infusion pump of small dimensions and low weight allowed for a most flexible administration of OHCbl in everyday life. Treatment was started with OHCbl 1 mg three times a week and increased to daily administration, resulting in tHcy concentrations in plasma within the target range (< 60 µmol/l [2]) (Fig. 1a).

Patient 1 is now 3 years of age and shows a normal psychomotor development. Except for hyperopia she has no signs and symptoms of cblC deficiency.

### Patient 2

Patient 2, a girl, was born after 37 weeks of gestation by Caesarian section due to placenta praevia and intrauterine growth retardation (birth weight 2240 g). She is the second child (G3P2) of healthy, non-consanguineous parents of Caucasian origin. She presented with respiratory distress after birth during the first hour of life. Newborn screening was unremarkable, but did not comprise remethylation disorders. A persistent ductus arteriosus Botalli required surgical occlusion at the age of 5 months. Patient 2 showed a severe failure to thrive, muscular hypotonia, developmental delay, and macrocytic anemia at 9 months of age. Diagnostic work-up revealed a markedly elevated concentration of tHcy (180 µmol/l; reference range < 12) and low concentration of methionine (8 µmol/l; reference range 15–35). OHCbl (1 mg IV) was given, and the patient was transferred to our metabolic department for further work-up. At admission, the concentration of tHcy had dropped to 105 µmol/l. MMA in plasma was within the normal range (130 nmol/l; reference range 73–271). The pattern of acylcarnitines in plasma was unremarkable. Clinical work-up of the patient demonstrated epileptic encephalopathy, macular dystrophy of both eyes, and nystagmus. With clinical and biochemical findings being highly indicative for a cobalamin-related remethylation disorder, intravenous treatment with OHCbl 1 mg IV per day was continued and oral treatment with betaine (250 mg/kg per day) and folic acid (10 mg per day) was added. With this treatment, the concentration of tHcy dropped to 53 µmol/l (Fig. 1b). Sequence analysis revealed two compound heterozygous variants (c.904 + 469 T > C and c.923A > C, p.(Gln308Pro)) in the *MTRR* gene leading to the diagnosis of cblE disease. The variant c.904 + 469 T > C in intron 6 is the most common disease-causing variant [15, 16]. The variant c.923A > C which is absent from the reference population database gnomAD [17] has not been described as disease-causing so far. In silico analyses predict a deleterious effect on the gene or gene product [18, 19]. In accordance to the ACMG criteria, the variant was classified as variant of uncertain significance (VUS) [13].

With regard to long-term treatment, the parents were very reluctant to tolerate routine IM injections of OHCbl in their child. To avoid the burden of frequent IM injections, SQ administration of 1 mg OHCbl was initiated using a subcutaneous catheter device together with a portable infusion pump. The placement of the catheter and application of OHCbl infusions are readily accomplished by the parents at home and well tolerated by the patient. No adverse events occurred. The concentrations of tHcy remained stable with infusion intervals up to 3 days (Fig. 1b). Weekly intervals resulted in an increase of tHcy concentration in plasma above 60 µmol/l.

Patient 2 is now 3 years old. An evaluation at 34 months of age revealed a global developmental delay with a developmental age of 12 months. Furthermore, she suffers from ataxia and a severe feeding disorder. She receives intensive physiotherapy, occupational and speech therapy. The blood smear shows no evidence of macrocytic anemia.

### Patient 3

Patient 3, a girl, is the first child of healthy, consanguineous parents. She was born at term in Turkey. During the first months of life, developmental delay and microcephaly were recognized. She was presented to our department at the age of 8 months. At presentation, concentration of tHcy was markedly elevated (164 µmol/l; reference range < 12). Mutation analysis revealed a homozygous pathogenic variant in the *MMACHC* gene (c.394C > T, p. Arg132Ter), confirming the diagnosis of cblC deficiency. The patient is now 16 years old. She suffers from severe mental retardation, multifocal epilepsy, hyperkinetic movement disorder, astigmatism, hyperopia, strabismus, and QTc elongation.

Since diagnosis, she has been treated with OHCbl, folic acid (7.5 mg per day), and betaine (up to 6 g per day). During childhood, concentrations of tHcy in plasma were largely stable (mean 58 µmol/l) under treatment with OHCbl 1 mg IM every other week given by the local pediatrician. However, starting at 12 years of age, tHcy concentrations in plasma increased up to 92 µmol/l (Fig. 1c), and treatment was adjusted gradually. Weekly administration of OHCbl 1 mg IM still resulted in tHcy concentrations above the target range (< 60 µmol/l) [2]. As the patient did not tolerate IM injections at a higher frequency or higher injection volumes, the administration was changed to SQ. OHCbl was administered via a SQ catheter and a portable infusion pump at home by the parents. The pump-guided SQ administration was well tolerated. No complications occurred. Substitution of OHCbl 1 mg SQ three times a week finally resulted in plasma tHcy concentrations within the target range (Fig. 1c).

### Patient 4

Patient 4, a boy, was born at term (birth weight 3610 g). He is the second child (G2P2) of healthy, non-consanguineous parents of Caucasian origin. Postnatal adaptation was uneventful. Newborn screening was collected at 3 days of age and was unremarkable, but did not comprise remethylation disorders. At 3 months of age, the patient was admitted to hospital due to progressive muscular hypotonia and drowsiness. He presented as floppy infant with little motor activity. Laboratory investigations showed neutropenia and megaloblastic anemia requiring transfusion. Electroencephalography revealed encephalopathy. Magnetic resonance imaging showed dilated lateral ventricles, hypoplasia of the corpus callosum, cerebellar hypoplasia, and a small bridging vein thrombosis. Subsequent metabolic workup demonstrated a markedly elevated concentration of tHcy (179 µmol/l; reference range < 12) and a decreased concentration of methionine (2 µmol/l; reference range 9–42) in plasma with normal concentrations of propionylcarnitine and MMA. These findings are highly indicative for a cobalamin-related remethylation disorder, thus OHCbl 1 mg IM, folic acid 20 mg PO, betaine 200 mg/kg PO, and methionine 50 mg PO per day were started. With this treatment, tHcy in plasma declined to 42 µmol/l (Fig. 1d) and methionine normalized. The diagnosis of cblG deficiency was confirmed by sequence analysis of the *MTR* gene. Two variants, the pathogenic variant c.3395G > A, p.Arg1132Gln [20] and the variant c.250-1G > C, p.? were identified in compound heterozygous state. The variant c.250-1G > C has not been described before (HGMD 2020.4) but was classified as (likely) pathogenic. It affects the canonical splice acceptor site of exon 3 resulting in either a frameshift with premature translational arrest, or exon skipping.

Within the following weeks, treatment was adjusted based on tHcy concentrations in plasma (Fig. 1d). To achieve tHcy concentrations as low as possible (< 40 µmol/l), 1 mg OHCbl every 3 weeks was sufficient. However, at the age of 31 months, these IM injections became a massive burden for the patient. He vigorously struggled against each injection. Both the parents and the home care nurse considered IM injections not to be acceptable any longer. Based on our previous experiences, we started SQ administration of OHCbl using an infusion pump. Patient 4 tolerated the placement of the SQ catheter and the infusion very well. Both could be executed by the parents during everyday life. Treatment was started with OHCbl 1 mg one time a week resulting in tHcy concentrations within the target range (< 60 µmol/l [2]) (Fig. 1d). An increase in administrations did not result in a further decrease of tHcy concentrations.

The patient is now 4 years of age. He has been diagnosed with a neurodevelopmental and developmental coordination disorder and requires occupational and physical therapy. At the age of 6 months he developed hydrocephalus communicans and a ventriculoperitoneal shunt had to be placed. His blood count normalized within days and stayed normal.

## Discussion

The treatment of cobalamin-related remethylation disorders requires the lifelong parenteral administration of OHCbl. To date, this is mainly achieved by routine IM injections. The dosage and frequency of OHCbl administration are titrated in each individual patient to the minimum effective dose in order to account for the painful injections. This may result in undertreatment, a possible risk factor for disease progression and the occurrence of complications [21].

We describe the parenteral administration of OHCbl using a SQ catheter system and a portable infusion pump in two patients with cblC, one patient with cblG, and one patient with cblE defect. To our knowledge, this is the first report of a pump-controlled SQ OHCbl infusion in patients with combined and isolated remethylation disorders.

All our patients required IM administrations of OHCbl that were not or no longer accepted by the patients and/or their parents regarding frequency or way of administration at some point of the disease course. The placement of a SQ catheter is an easy, almost painless procedure, which requires minimal medical skills. It was well tolerated by the patients and could readily be accomplished by the parents at home. In contrast, IM injections executed by the parents were not conceivable at any point. Once placed, the catheter device could be used for up to 3 days [22]. We did not encounter any adverse events such as infections of the catheter site or local infusion reactions. The slow SQ administration of OHCbl using an infusion pump prevented the pain induced by the swift delivery of the medication in IM or SQ injections. The use of a portable syringe driver pump of small dimensions and low weight allowed for a most flexible administration of OHCbl in everyday life e.g. during sleep, kindergarten, or school. All these aspects allowed for an individual OHCbl dosing at the required frequency and thus improving metabolic control in terms of tHcy concentrations within the target range or even continuing therapy [2].

The efficacy of SQ OHCbl administration in remethylation disorders is still under debate. In general, medications administered SQ have been found to be equivalent in efficiency when compared to other routes such as IV or PO [12]. OHCbl in particular is an ideal medication for subcutaneous administration on the basis of water solubility, neutral pH, and low viscosity [23]. A study on cblC patients found the biochemical effect of SQ administration to be identical to IM administration after more than 1 year of follow-up [9]. Another study suggested the SQ route to be less effective when compared to IM injections in an adult cblC patient [4].

Although the efficacy of a single SQ injection of OHCbl compared to a single IM injection of OHCbl may not be clear, the use of a SQ catheter enables more frequent dosing without further punctures and thus might improve metabolic control. These assumptions are in line with reports on patients suffering from cblA methylmalonic aciduria [11] and methylmalonic aciduria due to methylmalonyl-CoA mutase [10] who have been effectively treated using a SQ catheter for OHCbl injections.

As noted above, the pain and discomfort caused by both IM and SQ injections is not only related to the punctures, but also to the swiftly delivered volume of the medication, which is mostly 1 ml per 1 mg of OHCbl. As a consequence, IM and SQ administration of OHCbl is often limited to 1 mg. However, in addition to an increased frequency, a dose escalation might be effective to improve metabolic control in Cbl patients [9, 24–26]. The slow infusion of OHCbl using a pump allows for higher doses without discomfort or pain. As OHCbl has been shown to be stable in isotonic sodium chloride at room temperature up to 1 month [27], it may be administered at higher doses over several hours, if desired. Hence, this route of administration may provide a feasible possibility for treatment optimization studies [24].

The patients described in our report had an early onset disease phenotype or were diagnosed by newborn screening. In view of the fact that our approach is safe, effective, and acceptable for infants, toddlers, and severely affected patients, we also expect it to be a convenient alternative for juvenile and adult onset phenotypes.

Most of the knowledge about the management of remethylation disorders is derived from the experience with individuals with the cblC defect, the most frequent remethylation disorder [2]. In clinical practice, the rarer isolated as well as combined remethylation disorders are generally managed identically [2]. Thus, our experiences derived from patients with cblC, cblG, and cblE may be translated to all cobalamin-related remethylation disorders.

## Conclusions

In conclusion, pump-controlled infusion of OHCbl via a SQ catheter in cobalamin-related remethylation disorders is safe, effective, and acceptable. The strength of the described SQ infusion therapy lies within the ease of use requiring minimal medical and technical skills and thus enabling parents to administer their children’s medication independently at home even in small neonates, children, and severely impaired patients. It provides a large flexibility and minimal interference with daily life. The almost painless access and way of administration alleviates the burden of disease and promotes adherence to therapy.

## Methods

### Patients

We report the data of four patients with cobalamin-related remethylation disorders (two patients with cblC (OMIM #277400), one patient with cblG (OMIM #250940), and one patient with cblE (OMIM #236270)) followed at our metabolic center. Data of the patients are reported anonymized. The study is in accordance with the guidelines of the local ethics committee. Written informed consent for publication of clinical details was obtained from the patients’ parents.

### Biomarker analyses

Filter paper samples from newborn screening were analyzed by MS/MS as previously described [28–32]. For analysis of acylcarnitines in plasma, blood samples were collected in tubes prepared with lithium-heparin. Plasma was separated by centrifugation within 1 h after sampling. Analysis was performed using electrospray ionization-tandem mass spectrometry (ESI–MS/MS) [28, 33, 34]. Organic acids were analyzed in urine using capillary gas chromatography followed by mass spectrometry as described elsewhere [35]. Vitamin B12 was measured using a Cobas electrochemiluminescence immunoassay (ECLIA), homocysteine using an enzymatic assay (Roche Diagnostics, Mannheim) following the manufacturer’s instructions. Reference values are given according to the laboratories’ and manufacturers’ experience, respectively. MMA in plasma and urine were determined using gas chromatography (GC)-MS [36, 37].

### Molecular analyses

Molecular analysis of the *MMACHC* (GenBank: NM_015506.3), *MTRR* (GenBank: NM_002454.2), and *MTR* gene (GenBank: NM_000254.2) was performed by Sanger sequencing [14, 32, 38].

### Administration of vitamin B12 by subcutaneous infusion

1 mg of OHCbl, diluted in 2 ml isotonic saline solution, was administered via the Cleo® (teflon cannula, 27 gauge/6 mm) 90 6/60 SQ infusion catheter system (Smiths medical, Grasbrunn) or Soft Glide® (steel cannula, 27 gauge/6 mm) SQ catheter set (THM, Duisburg). For controlled infusion over 1 h the portable CRONO S-PID infusion pump (LICHER MT GmbH, Wedemark) was used. The SQ catheters were changed according to the manufacturer’s recommendations every 2–3 days. Parents were educated to place the catheter and use the infusion pump independently at home.

## Data Availability

All data generated or analyzed during this study are included in this published article.

## Abbreviations

tHcy: Total homocysteine
MMA: Methylmalonic acid
cbl: Cobalamin
OHCbl: Hydroxycobalamin
MS/MS: Tandem mass spectrometry
IV: Intravenous
IM: Intramuscular
SQ: Subcutaneous
PO: Per os
QW: Weekly
Q2W: Every 2 weeks
Q3W: Every 3 weeks
BIW: Twice a week
TIW: Three times a week
FIW: Five times a week
QD: Daily
E5D: Every 5 days
